# Investigation of Thrombosis Volume, Anticoagulants, and Recurrence Factors in Portal Vein Thrombosis with Cirrhosis

**DOI:** 10.3390/life10090177

**Published:** 2020-09-04

**Authors:** Tsuyoshi Suda, Hajime Takatori, Takehiro Hayashi, Rika Horii, Kouki Nio, Takeshi Terashima, Noriho Iida, Masaaki Kitahara, Tetsuro Shimakami, Kuniaki Arai, Taro Yamashita, Tatsuya Yamashita, Eishiro Mizukoshi, Masao Honda, Kenichiro Okumura, Kazuto Kozaka, Shuichi Kaneko

**Affiliations:** 1 Department of Gastroenterology, Kanazawa University Graduate School of Medical Science, Kanazawa, Ishikawa 920-8641, Japan; t.suda1112@gmail.com (T.S.); hayassy@live.jp (T.H.); rhorii@m-kanazawa.jp (R.H.); niokouki2000@gmail.com (K.N.); tera20jp@gmail.com (T.T.); niida@m-kanazawa.jp (N.I.); mkitahara2007@gmail.com (M.K.); shimakami@m-kanazawa.jp (T.S.); arai@m-kanazawa.jp (K.A.); taroy62m@staff.kanazawa-u.ac.jp (T.Y.); ytatsuya@m-kanazawa.jp (T.Y.); eishirom@m-kanazawa.jp (E.M.); mhonda@m-kanazawa.jp (M.H.); skaneko@m-kanazawa.jp (S.K.); 2 Department of Radiology, Kanazawa University Graduate School of Medical Science, Kanazawa, Ishikawa 920-8641, Japan; k.okumura223@gmail.com (K.O.); kosaka2007@gmail.com (K.K.)

**Keywords:** portal vein thrombosis, cirrhosis, anticoagulation, volume, reduction, treatment efficiency, recurrence, Child–Pugh, FIB-4, platelet

## Abstract

This retrospective study investigated factors influencing the portal vein thrombosis (PVT) volume and recurrence in 52 cirrhosis patients with PVT from November 2008 to September 2018. All patients were treated with danaparoid sodium with or without additional antithrombin III. Blood platelet counts significantly correlated with the PVT volume (r^2^ = 0.17; *P* < 0.01). Computed tomography confirmed recurrence as PVT aggravation was reported in 43 patients, with ≥50% PVT volume reduction following anticoagulation therapy. In 43 patients, recurrence significantly correlated with the pretreatment PVT volume (*P* = 0.019). Factors influencing recurrence included a Child–Pugh score >8 (*P* = 0.049) and fibrosis index ≤7.0 based on four factors (FIB-4) (*P* = 0.048). Moreover, the relationship between recurrence and correlating factors showed that 15 patients who received warfarin experienced recurrence more often when Child–Pugh scores were >8 (*P* = 0.023), regardless of maintenance treatment. For patients who did not receive warfarin, a PVT volume ≥3.0 mL significantly influenced recurrence (*P* = 0.039). Therefore, the platelet count influences the PVT volume. The pretreatment PVT volume correlated with recurrence after anticoagulation therapy. According to the Kaplan–Meier curve, risk factors for PVT recurrence after anticoagulation therapy included Child–Pugh scores >8 and FIB-4 ≤7.0. Therefore, the FIB-4 is a unique factor that shows trends opposing other liver function markers.

## 1. Introduction

Portal vein thrombosis (PVT) is defined as the presence of a thrombus in the lumen of the vein that causes complete or partial obstruction of portal venous blood flow. If a thrombus is present, then a cavernoma is evidence of chronic thrombosis. Extrahepatic portal vein obstruction includes occlusion of the portal vein, with or without thrombosis, and cavernoma formation, with or without portal hypertension [1].

PVT can occur in various clinical situations such as cirrhosis, myeloproliferative disease, cancer, and infection [2]. Common thrombophilia also has a role in PVT [3]. PVT is related to a previous decrease in portal blood flow velocity [3]. This condition is known to occur with the progression of liver diseases such as advanced cirrhosis [4,5]. The estimated prevalence of PVT in patients with cirrhosis ranges from 0.6 to 26% [6]. However, the cumulative incidence rates span 4.6% to 12.8% [7,8,9], 10.2% to 20% [7,8], and approximately 38.7% [7,8] at 1, 5, and 8 to 10 years, respectively. Furthermore, PVT reportedly increases the risk of liver decompensation and risk of mortality in patients with cirrhosis [10,11].

Generally, patients treated with anticoagulants have significantly higher rates of PVT recanalization than patients not treated with anticoagulants. Furthermore, PVT recanalization rates were 71% with anticoagulants and 42% without anticoagulants. Complete PVT recanalization rates were 53% with anticoagulants and 33% without anticoagulants. Additionally, the PVT progression rate decreased significantly for patients using anticoagulants compared to those not using anticoagulants. There was no difference in the bleeding rates of patients using and not using anticoagulants [12].

Danaparoid sodium is an effective anticoagulant that inhibits activated factor Xa. An important feature of this agent is the low rate of cross-reactivity with antibodies associated with immune-mediated heparin-induced thrombocytopenia [13]. This agent is also unlikely to cause gastrointestinal hemorrhage, making it more effective and safer than heparin [14]. Danaparoid sodium is safe and effective for the treatment of PVT in patients with liver diseases [15,16]. Antithrombin III (AT-III) is also an essential treatment for PVT patients with a low concentration of AT-III [17,18].

Previously, we reported danaparoid sodium therapy with and without AT-III anticoagulation therapy for PVT in cirrhosis patients [19]; in that report, we presented the efficacy and safety of danaparoid and found that the prognosis for PVT patients depended on the hepatic reserve capacity. However, the recurrence of PVT was high. PVT with cirrhosis and a history of resolved PVT are considered risk factors for recurrent PVT [7,9]. Currently, detailed information surrounding these risk factors is limited. Therefore, in the present study, we focused on the PVT volume, PVT reduction, and PVT recurrence. We also performed further analyses of some patients from our previous study and investigated their related factors.

## 2. Results

### 2.1. Patient Characteristics

Table 1 shows the clinical characteristics of patients with PVT before treatment. All the patients were diagnosed with cirrhosis. Many had hepatocellular carcinoma (HCC) (40.4%), and approximately half had a history of treatment for varices (48.1%). Most patients were male (76.9%). The rate of PVT, including thromboses in the main trunk of the portal vein (MPV), was 82.7%. The median Child–Pugh score, albumin–bilirubin (ALBI) score, and fibrosis based on the four clinical factors (FIB-4) index was 8, −1.8, and 7.6, respectively. The median PVT volume was 3.2 mL.

### 2.2. Relationship between Correlation Factors and the PVT Volume

**Figure 1:**
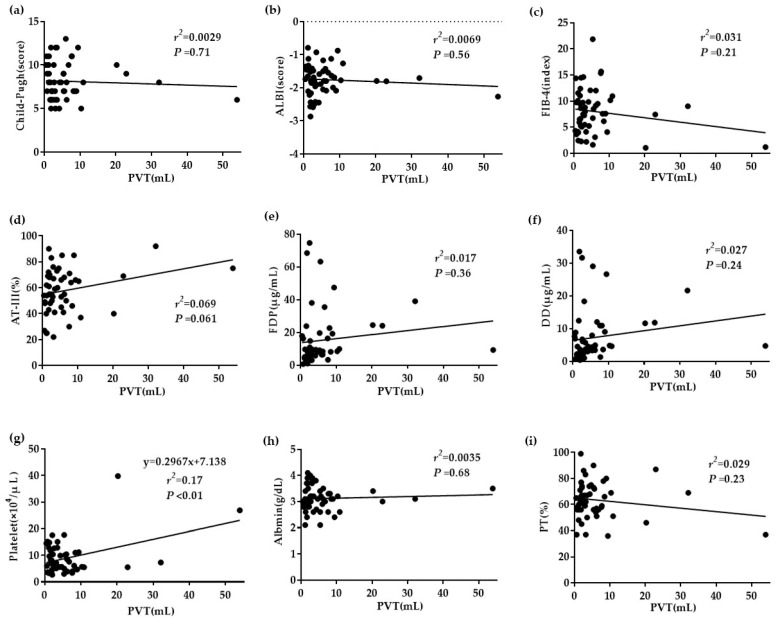
The relationship between correlation factors and the portal vein thrombosis (PVT) volume. (**a**–**c**) No significant differences in the Child–Pugh score, albumin–bilirubin (ALBI) score, or fibrosis index ≤7.0 based on four factors (FIB-4) index. (**d**–**f**) No significant differences in plasma antithrombin III (AT-III), plasma fibrinogen degradation products (FDP), or plasma D dimer (DD). (**g**) Blood platelet counts correlated significantly with the PVT volume (r^2^ = 0.17; *P* < 0.01). (**h**,**i**) No significant differences in serum albumin and plasma PT activity.

An analysis of the 52 patients indicated a significant correlation between blood platelet counts and the PVT volume (r^2^ = 0.17; *P* < 0.01). However, other factors, such as the Child–Pugh score, ALBI score, FIB-4 index, plasma AT-III level, plasma fibrinogen degradation products (FDP) level, plasma D dimer (DD) level, serum albumin level, and plasma prothrombin time (PT) activity, revealed no significant differences (Figure 1).

### 2.3. Relationship between Correlation Factors and PVT Reduction

**Figure 2:**
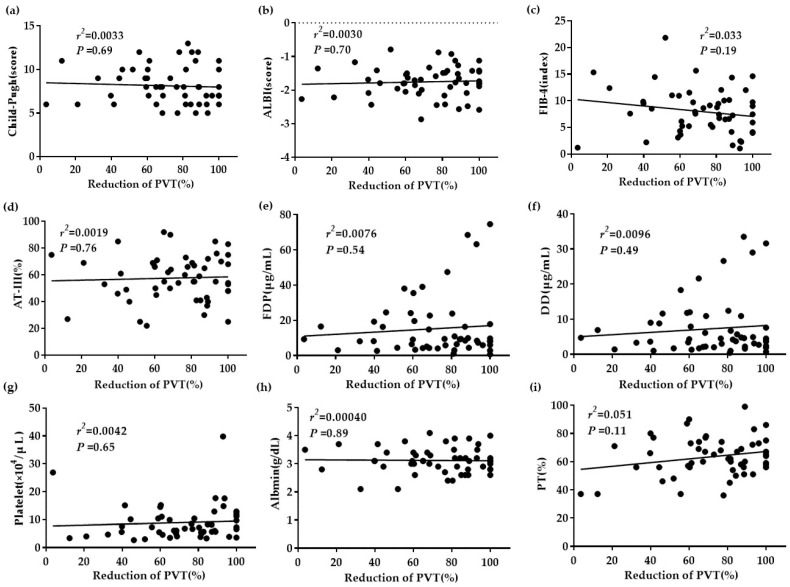
The relationship between correlation factors and PVT reduction. (**a**–**i**) No significant differences in any of the presented factors.

An analysis of the 52 patients indicated no significant difference in the correlation between PVT reduction and the Child–Pugh score, ALBI score, FIB-4 index, plasma AT-III level, plasma FDP level, plasma DD level, blood platelet count, serum albumin level, or plasma PT activity (Figure 2).

**Figure 3:**
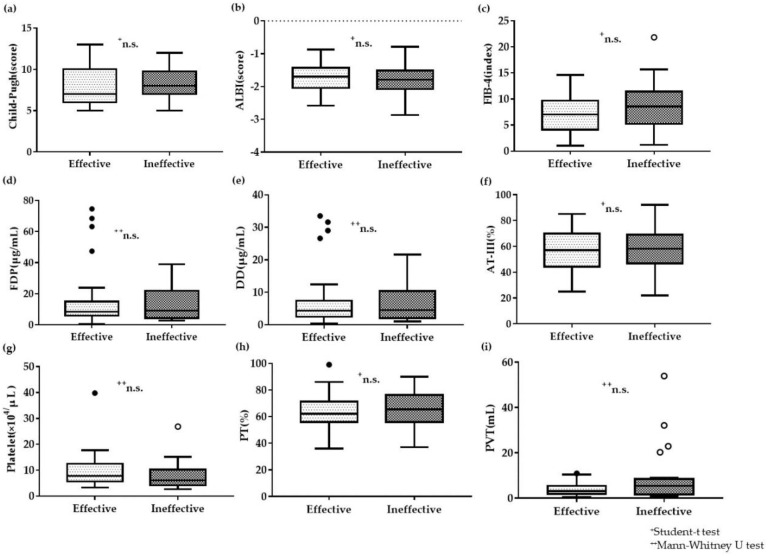
Correlation factors involved in treatment efficacy for the effective and ineffective groups (**a**–**i**) No significant differences in any of the presented factors. ^+^ Student’s t test, ^+^^+^ Mann–Whitney U test.

Next, the effective group (n = 28) was defined as a reduction of ≥75% in the volume of PVT. Ineffective group (n = 24) was considered if the reduction in the PVT volume was <75%. An analysis was performed in those two groups to investigate the correlation factors that affected treatment efficacy. However, this analysis also indicated no significant difference in the correlation factors: the Child–Pugh score, ALBI score, FIB-4 index, plasma AT-III level (this date has been presented in our previous report [19]), plasma FDP level, plasma DD level, blood platelet count, plasma PT activity, or pretreatment PVT volume (Figure 3). In addition, serum albumin level and total serum bilirubin also revealed no significant difference (Supplementary Figure S1).

### 2.4. Correlation Factors Affecting Recurrence after Anticoagulation Therapy

**Figure 4:**
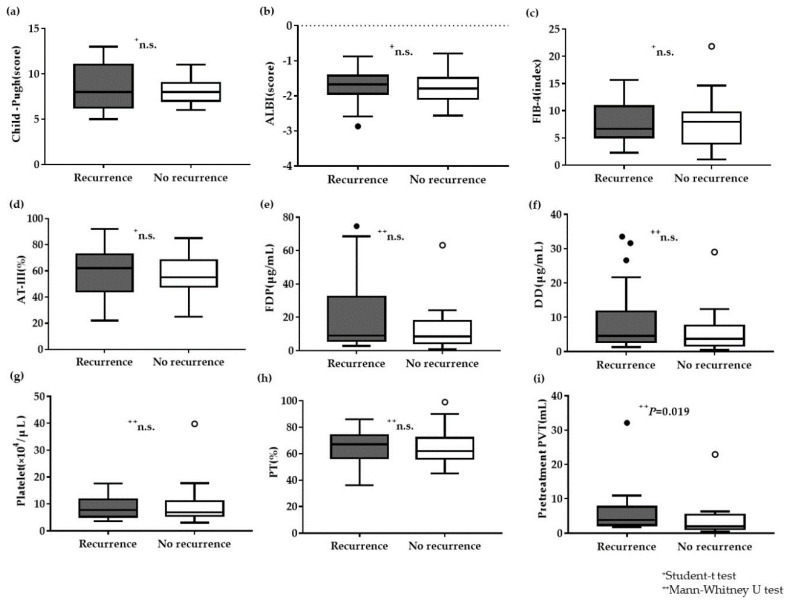
Correlation factors affecting recurrence after anticoagulation treatment. (**a**–**c**) No significant differences in the Child–Pugh score, ALBI score, or FIB-4 index. (**d**–**h**) No significant differences in the plasma AT-III, FDP, DD, blood platelets, or plasma PT activity. (**i**) Pretreatment volume of PVT is significantly greater in the recurrence group than in the no recurrence group (*P* = 0.019). ^+^ Student’s t-test, ^+^^+^ Mann–Whitney U test.

An analysis of the correlation factors affecting recurrence in 43 patients revealed no significant differences in the following factors: Child–Pugh score, ALBI score, FIB-4 index, plasma AT-III level, plasma FDP level, plasma DD level, blood platelet counts, and plasma PT activity (Figure 4). Additionally, analysis of the reduction of PVT, serum albumin level, age, total serum bilirubin, serum aspartate transaminase (AST) level, and serum alanine aminotransferase (ALT) level also showed no significant differences (Supplementary Figure S2). Only the post-treatment PVT volumes demonstrated a significant difference between the recurrence and pretreatment PVT volumes (*P* = 0.019) (Figure 4).

Factors, such as sex, HCC diagnosis, previous splenectomy or partial splenic embolization (PSE), history of varices treatment, and previous PVT (including thrombosis in the MPV) showed no significant differences. A summary of these results is presented in Table 2. As for pretreatment PVT volume, this was also confirmed as a significant correlation factor (*P* = 0.017) limited to the effective group (Supplementary Table S1).

### 2.5. Recurrence Events after Anticoagulation Therapy

To investigate the recurrence events among the 43 patients, we divided the correlation factors into two groups. The cut-off values for both groups were decided based on the receiver-operating characteristic (ROC) curve and the median. The following factors were below: Child–Pugh score ≤8 (n = 28) or >8 (n = 15); ALBI ≤−1.7 (n = 20) or >−1.7 (n = 23); FIB-4 ≤ 7.0 (n = 20) or >7.0 (n = 23); plasma AT-III ≥60 (n = 22) or <60 (n = 21); blood platelet count ≥8.0 × 10^4^ (n = 19) or <8.0 × 10^4^ (n = 24); PVT volume ≥3.0 mL (n = 20) or <3.0 mL (n = 23); history of splenectomy or PSE present (n = 7) or absent (n = 36); history of varices treatment present (n = 20) or absent (n = 23); and HCC diagnosis present (n = 16) or absent (n = 27). The Kaplan–Meier curve was used to analyze the recurrence event for each correlation factor.

**Figure 5:**
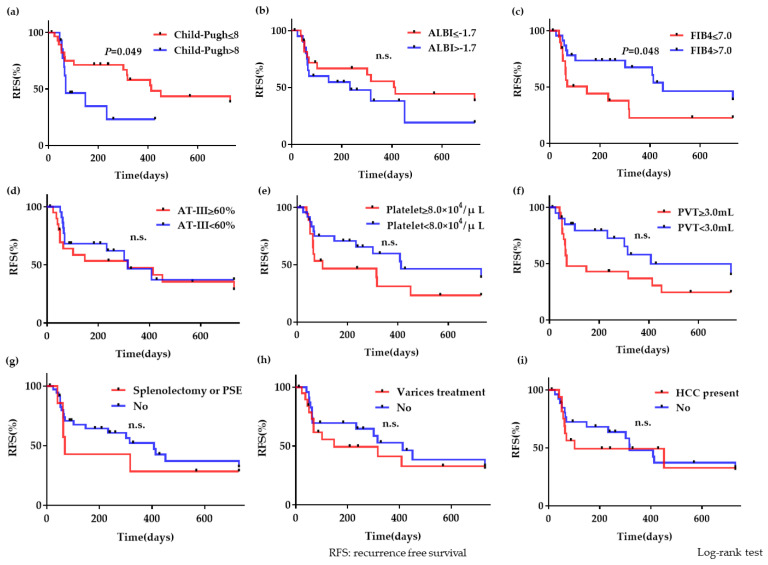
Recurrence events after anticoagulation treatment (**a**) More recurrence events occurred in the group with Child–Pugh scores >8 than in the group with Child–Pugh scores ≤8, and the difference was statistically significant (*P* = 0.049). (**b**) However, there were no significant differences between the group with ALBI ≤−1.7 and that with ALBI >−1.7. (**c**) More recurrence events occurred in the group with the FIB-4 index ≤7.0 than in the group with the FIB-4 index >7.0, and the difference was statistically significant (*P* = 0.048). (**d**–**f**) There were no significant differences between groups with plasma AT-III ≥60% or <60, blood platelet count ≥8.0 × 10^4^/μL or <8.0 × 10^4^/μL, and the pretreatment PVT volume ≥3.0 mL or <3.0 mL. (**g**–**i**) There were no significant differences between groups with the presence or absence of previous splenectomy or partial splenic embolization (PSE), presence or absence of varices treatment history, and presence or absence of hepatocellular carcinoma (HCC) diagnosis.

More recurrence events occurred in the group of patients with Child–Pugh scores >8 than in the group with Child–Pugh scores ≤8 (hazard ratio, 2.79; 95% confidence interval, 1.006–7.734; *P* = 0.049), and in the group with FIB-4 ≤7.0 than in the group with FIB-4 >7.0 (hazard ratio, 2.35; 95% confidence interval, 1.007–5.497; *P* = 0.048). These outcomes were statistically significant. Other correlation factors revealed no significant differences (Figure 5).

### 2.6. Recurrence Events with or without Maintenance Treatment

We analyzed the relationship between the recurrence events and correlation factors, with or without maintenance treatment, using the Kaplan–Meier curve. Fifteen patients who received warfarin were classified as “with maintenance treatment” and 17 patients who did not receive warfarin were classified as “without maintenance treatment.” None of the parameters, including the Child–Pugh score, ALBI score, FIB-4 index, blood platelets, plasma AT-III, and volume of pretreatment PVT, demonstrated any significant difference in patients with maintenance treatment and patients without maintenance treatment (Supplementary Table S2).

**Figure 6:**
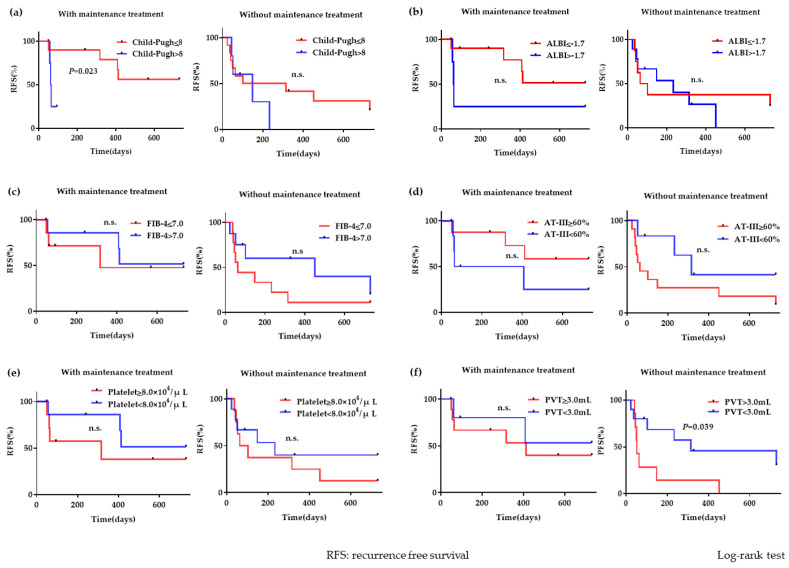
PVT recurrence with or without maintenance treatment (**a**) The group of patients with maintenance treatment experienced more recurrence events when Child–Pugh scores were >8 than when Child–Pugh scores were ≤8; this difference was statistically significant (*P* = 0.023). There was no significant difference between groups with and without maintenance treatment. (**b**–**e**) No significant differences were found between the groups with ALBI ≤−1.7 and >−1.7, FIB-4 ≤7.0 and FIB-4 >7.0, plasma AT-III ≥60% and <60%, or blood platelet ≥8.0 × 10^4^/μL and <8.0 × 10^4^/μL. (**f**) A pretreatment PVT volume ≥3.0 mL was a statistically significant factor for recurrence (*P* = 0.039) in the group without maintenance treatment. The PVT volume with maintenance treatment was not statistically significant.

The group of patients with maintenance treatment experienced more recurrence events when the Child–Pugh scores were >8 (n = 5) than when the Child–Pugh scores were ≤8 (n = 10); this difference was statistically significant (hazard ratio, 13.75; 95% confidence interval, 1.438–131.6; *P* = 0.023). However, there was no significant difference between the groups of patients with and without maintenance treatment. A PVT volume ≥3.0 mL (n = 7) significantly predicted recurrence (hazard ratio, 3.745; 95% confidence interval, 1.07–13.1; *P* = 0.039) when patients did not receive maintenance treatment. Nonetheless, there was no significant difference in the PVT volume of the group of patients with maintenance treatment. Other correlation factors revealed no statistical significance (Figure 6).

## 3. Discussion

In this study, all the PVT cases were treated with danaparoid sodium-based treatment with or without AT-III for 2 weeks. There are several reports in which patients received anticoagulant treatment for at least 6 months [20,21]. On the other hand, previous studies conducted in Japan have reported treatment efficiency with short-term administration of danaparoid sodium for only 2 weeks [15,19]. The short duration of treatment is directly dependent on the health care system and social background of people in Japan. However, despite 2 weeks being a considerably short time frame, we considered that it was acceptable because danaparoid sodium-based treatment revealed a high reduction rate of PVT (72.0–77.3%) within this short duration [15,19]. Considering the short duration of treatment, the patients were followed-up regularly after treatment and danaparoid sodium was re-administered if PVT recurrence or aggravation was confirmed. As for maintenance treatment, patients on the waiting list for liver transplant were recommended anticoagulant treatment. However, bleeding events, including gastrointestinal bleeding occurred with a certain probability, and, therefore, maintenance treatment was considered in only selected cases [22,23]. Thus, we concluded that PVT maintenance treatment was not necessary for all the patients and that it should be reserved only for selected patients who received insufficient anticoagulant treatment or had risk factors.

According to the analysis, the PVT volume correlated significantly only with blood platelet count, although the correlation was very weak (*P*^2^ = 0.17; *P* < 0.01). Other factors did not show a significant relationship. Thus, we considered the possibility of a correlation between blood platelet count and PVT volume. Some reports indicated that the increased risk of PVT in cirrhosis is associated with decreased liver function (Child–Pugh score B/C) [7], low serum albumin [24], and increased prothrombin time [25]. However, these factors had no significant impact on the PVT volume in our study. Blood platelet count is known to be an important factor in PVT occurrence. Therefore, a low platelet count is predictive of PVT development in cirrhosis patients [26].

However, for cirrhosis patients who had undergone splenectomy, the incidence of PVT after surgery was 37.7% (49/130), and an increased platelet count has been described as a risk factor [25]. Another report also revealed that the platelet count was significantly higher in the PVT group (mean platelet count, 399.3 × 10^9^/L) than in the non-PVT group (mean platelet count, 296.9 × 10^9^/L) after laparoscopic splenectomy [27]. Another report indicated that 16.9% (71/420) of cirrhosis patients experienced PVT after splenectomy. The risk factor was postoperative thrombocytosis (platelet count ≥200,000/μL) on day 7 [28]. Furthermore, PVT has also been reported as an adverse event with the use of thrombopoietin (TPO) agonists. TPO agonists elevate platelets for longer periods than transfusions without increasing portal pressure. However, eltrombopag, one of the TPO agonists, caused PVT when platelets inadvertently increased dramatically in a small number of patients [29].

Based on these reports, low platelet levels are usually related to PVT because they can reflect low liver function and/or the presence of portal hypertension, causing splenomegaly. However, a drastic increase in platelets such as after splenectomy or with the use of TPO agonists is also a risk factor for PVT.

Although the platelet count may influence the PVT volume, it does not influence the PVT reduction after anticoagulation treatment. To our knowledge, no report has investigated the relationship between the PVT volume and platelet count.

Factors that contributed to therapeutic efficacy such as sustained liver function (Child–Pugh class A) have been previously reported [30]; however, we could not find significant factors. The Child–Pugh score was not a significant factor in anticoagulation therapy in this study.

It is vital to investigate predictive factors when anticoagulant drugs are used to treat PVT. One factor affecting recurrence after anticoagulation therapy is the pretreatment PVT volume. As previously stated, there is no obvious factor that predicts PVT recurrence. However, it is reasonable to conclude that a large PVT volume tends to lead to recurrence. A diagnosis of HCC, previous splenectomy or PSE, previous treatment of varices, previous PVT including thrombosis of the MPV, and decreased liver function did not affect the recurrence of PVT.

According to the Kaplan–Meier curve for PVT recurrence after anticoagulation therapy, Child–Pugh scores >8 and the FIB-4 index ≤7.0 were considered recurrence factors (*P* < 0.05). Although the PVT volume affected recurrence after anticoagulation treatment in this study, our investigation of groups with PVT ≥3.0 mL and with PVT <3.0 mL using the Kaplan–Meier curve could not, unfortunately, prove a significant difference. However, another investigation using the Kaplan–Meier curve showed a significant difference between groups with PVT ≥3.0 mL and PVT <3.0 mL without maintenance treatment.

FIB-4 was a novel factor. The FIB-4 index has been known to be correlated with the stages of liver fibrosis [31,32,33]. The cutoff for diagnosing severe fibrosis is set at a low value, such as 1.45 for chronic hepatitis B patients [32] and as 1.30 for nonalcoholic fatty liver disease patients [33]. Although FIB-4 usually reveals liver fibrosis, our data showed frequent recurrence in the group with FIB-4 ≤7.0. This statement may appear counterintuitive. However, our cutoff of 7.0 was extremely high for diagnosing severe liver fibrosis. FIB-4 was calculated using the following formula: age × AST / Platelet count × √ALT. The platelet count is positively correlated with the PVT volume, and a larger PVT volume before treatment is a risk factor for recurrence. The platelet count appears to influence the decrease in the FIB-4 score. However, platelet count alone was not a significant recurrence factor and other factors (age, AST, ALT) also revealed no significant differences in the recurrence. There was a possibility that FIB-4 may indicate factors other than liver fibrosis. FIB-4 is a unique factor that shows trends opposing other liver function markers as a factor that influences recurrence after PVT treatment, and its value should be further investigated. We also evaluated the recurrence rates with or without maintenance treatment. More recurrence events occurred in the group with Child–Pugh scores >8 (*P* = 0.023) with maintenance treatment, and a PVT volume ≥3.0 mL was a significant factor for recurrence (*P* = 0.039) without maintenance treatment.

The prevention of PVT recurrence using anticoagulant agents was effective, leading to significant reductions in the risk of PVT development and liver decompensation, marked improvements in liver function and Child–Pugh score, and improvements in overall survival [10]. As discussed above, it is particularly recommended that patients with PVT who are on the waiting list for a liver transplant should undergo anticoagulation to reduce post-transplant mortality and morbidity [22,23]. However, there has been no consensus regarding the treatment strategy following the initial anticoagulant therapy. Our data indicated that maintenance therapy is recommended if the liver function is good (i.e., Child–Pugh score ≤ 8) and the pretreatment PVT volume is large (≥3.0 mL).

Several limitations existed in this study. First, this was a retrospective study; therefore, there may have been some confounding factors because there was no randomization. Second, four patients had exceptionally larger PVT volumes (≥20 mL) compared to others. Two of four patients had a recent treatment history of splenectomy or radiofrequency ablation and three of four patients had a post-treatment history of varices, which suggested high portal hypertension. As such, the large PVT volumes would have influenced the results of this study. Third, some patients had the benefit of AT-III, but others did not. We conducted a second analysis to confirm whether there was a difference in relation to factors and PVT volume, reduction, and recurrence. According to the results, the Child–Pugh score and the ALBI score were significantly higher in the group of patients treated with combination therapy than in the group treated with monotherapy. This result indicated that liver function was lower in patients with combination therapy before treatment. As PVT volume/reduction/recurrence, there were no significant differences in PVT volume (Supplementary Table S3).

Further research of the treatment regime for combined AT-III and danaparoid is required. We believe that our analysis is novel and has never been reported before. We hope that additional large-scale, long-term prospective studies will verify our results and clarify whether large PVT volumes affect liver function and prognosis, and the usefulness of the FIB-4 index to determine recurrence after anticoagulant therapy.

## 4. Materials and Methods

### 4.1. Study Design and Patients

We previously reported the aims and protocol of this study [19]. This retrospective study involved 52 cirrhosis patients with PVT treated with danaparoid sodium between November 2008 and September 2018. PVT was defined as the occurrence of thrombosis in the MPV, the first-order vessels of the left or right branch of the portal vein, or the superior mesenteric vein.

All PVT patients were treated with danaparoid sodium with AT-III (combination therapy) or without AT-III (monotherapy) at Kanazawa University Hospital. Combination therapy was performed from November 2008 to September 2011; monotherapy was performed from October 2011 to March 2014. Therefore, from April 2014 to September 2018, we performed combination therapy for patients with AT-III ≤70% and monotherapy for those with AT-III >70% (Table 1).

All patients with PVT included in this study received an intravenous injection of 1250 units of danaparoid sodium (Orgaran; MSD, Tokyo, Japan) twice daily for 2 weeks. This was the protocol treatment for PVT. Patients in the combination therapy group received an additional intravenous infusion of AT-III (Nonthron; Nihon Pharmaceutical, Tokyo, Japan) at a dose of 1500 units/day from days 1 to 5 and from days 8 to 12 (Supplementary Figure S3).

Maintenance treatment using warfarin after combination therapy or monotherapy was performed in certain patients. Drug indication was decided by the attending doctor taking into consideration the status of PVT, bleeding tendency, blood platelet count, coagulation markers, other comorbidities, and prognosis.

Recurrence was defined as the aggravation of PVT confirmed by computed tomography (CT) after anticoagulation therapy. Forty-three patients whose PVT volumes were reduced by ≥50% were selected for analysis because it was difficult to evaluate the recurrence of PVT in cases with <50% reduction. Patients’ medical records were reviewed and their demographic, clinical, laboratory, and imaging data were collected. All laboratory data were obtained on the first day±1day when combination therapy or monotherapy was administered.

The Institutional Review Board (IRB) of Kanazawa University Hospital approved the study’s treatment strategy and study protocol (no. 2016–096). The study was conducted according to the Declaration of Helsinki. The requirement for obtaining informed consent from the patients was waived by the IRB because of the study’s retrospective nature.

### 4.2. Measurement of PVT

PVT was confirmed by contrast-enhanced CT (CECT). We traced the thrombus on an axial CECT image and calculated the volume of the thrombus by using a three-dimensional image analysis system (Synapse Vincent Ver. 3 and Ver. 5; Fujifilm Medical Co., Tokyo, Japan). The measurement was performed by either a radiologist or a gastroenterologist.

CECT involved two stages: before anticoagulation therapy and between days 13 and 18 after treatment. PVT volume reductions were based on the following calculation: reduction of PVT (%) = {(PVT volume pretreatment − PVT volume post-treatment)/(PVT volume pretreatment)} × 100.

### 4.3. Statistical Analysis

Statistical analysis was performed using GraphPad Prism software 6.0 (GraphPad Software, San Diego, CA). Categorical variables were compared using the χ^2^ test or Fisher exact test when appropriate. The Student’s t-test or Mann–Whitney U test was used for continuous variables. Recurrence rates were analyzed using the Kaplan–Meier method with the log-rank test. All P values were two-tailed, and *P* < 0.05 was considered statistically significant.

## 5. Conclusions

During this analysis, we demonstrated that the PVT volume was significantly related to the platelet count and that the pretreatment PVT volume affected recurrence following anticoagulation treatment.

Child–Pugh scores >8 and the FIB-4 index ≤7.0 were factors that predicted recurrence after anticoagulation treatment. The FIB-4 index is a unique factor that shows trends opposing those of other liver function markers. Additionally, maintenance treatment was recommended if the liver function was good (Child–Pugh score of <8) and the pretreatment PVT volume was large (PVT volume ≥3.0 mL).

## Figures and Tables

**Table 1:** Patients’ characteristics.

	Patients (n = 52)
Age, years	64 (58, 70)
Sex, male, n (%)	40 (76.9)
Etiology, HCV/HBV/Alcohol, n (%)	28 (53.8)/6 (11.5)/8 (15.4)
HCC present, n (%)	21 (40.4)
Treatment history of splenectomy or PSE, n (%)	8 (15.4)
Treatment history of varices, n (%)	25 (48.1)
Including MPV, n (%)	43 (82.7)
Child–Pugh score	8 (5–10)
ALBI score	−1.8 (−2.1–−1.4)
FIB-4 index	7.6 (4.5–10.1)
Platelet (×10^4^/μL)	7.0 (5.0–11.2)
PT activity (%)	63 (56–73)
Albumin (mg/dL)	3.1 (2.8–3.4)
T-Bil (mg/dL)	1.3 (0.9–2.1)
AT-III (%)	57 (47–69)
FDP (μg/mL)	8.8 (4.6–18.9)
DD (μg/mL)	4.4 (2.2–9.0)
Pretreatment PVT (mL)	3.2 (1.9–6.8)

Median (IQR).

**Table 2:** Patients’ characteristics of the groups with and without recurrence.

	Recurrence (n = 24)	No Recurrence (n = 19)	*P*
Age, years	65 (58, 70)	64 (57, 73)	* n.s.
Sex, male, n (%)	18 (75)	15 (89)	^+^ n.s.
Etiology, HCV/HBV/Alcohol, n (%)	17 (71)/1 (4)/3 (3)	8 (38)/2 (11)/4 (21)	^+^ n.s.
HCC present, n (%)	9 (38)	7 (37)	^+^ n.s.
Treatment history of splenectomy or PSE, n (%)	5 (21)	2 (11)	^+^ n.s.
Treatment history of varices, n (%)	12 (46)	8 (47)	^+^ n.s.
Including MPV, n (%)	21 (88)	14 (74)	^+^ n.s.
Child–Pugh score	8 (6–11)	8 (7–9)	* n.s.
ALBI score	−1.7 (−1.9–−1.4)	−1.8 (−2.1–−1.5)	* n.s.
FIB-4 index	6.7 (5.1–10.1)	8.0 (4.0–9.7)	* n.s.
Platelet (×10^4^/μL)	7.8 (5.2–11.7)	6.8 (5.5–11.0)	** n.s.
PT activity (%)	67 (57–74)	62 (56–72)	* n.s.
Albumin (mg/dL)	3.1 (2.8–3.3)	3.1 (2.8–3.4)	* n.s.
T-Bil (mg/dL)	1.6 (0.9–2.5)	1.1 (0.9–1.5)	** n.s.
AT-III (%)	62 (47–72)	55 (47–69)	* n.s.
FDP (μg/mL)	9.0 (5.9–32.3)	8.4 (4.4–17.8)	** n.s.
DD (μg/mL)	4.6 (2.8–11.7)	3.7 (1.7–7.6)	** n.s.
Pretreatment PVT (mL)	3.8 (2.4–7.7)	2.0 (1.2–5.4)	** 0.019

Median (IQR); * Student‘s t-test; ** Mann–Whitney U test; ^+^ χ^2^ test or Fisher exact test.

## Supplementary Materials

The following materials are available online at https://www.mdpi.com/2075-1729/10/9/177/s1, Figure S1: Relationship between correlation factors and PVT reduction, Figure S2: Correlation factors affecting recurrence after anticoagulation therapy, Figure S3: Regimen of danaparoid sodium therapy with and without AT-III anticoagulation therapy, Table S1: Patients’ characteristics in the groups with and without recurrence in effective group, Table S2: Patients’ characteristics in the groups receiving and not receiving maintenance treatment, Table S3: Patients’ characteristics in the groups receiving monotherapy and combination therapy.

## Abbreviations

PVT	Portal vein thrombosis
AT-III	Antithrombin III
HCC	Hepatocellular carcinoma
MPV	The main trunk of the portal vein
CT	Computed tomography
CECT	Contrast-enhanced computed tomography
HCC	Hepatocellular carcinoma
FDP	Fibrinogen degradation products
DD	D dimer
PT	Prothrombin time
PSE	Partial splenic embolization
ALBI	Albumin–bilirubin
FIB-4	Fibrosis index based on four clinical factors
AST	Aspartate transaminase
ALT	Alanine aminotransferase
TPO	Thrombopoietin
