# Supplementary material for: Investigation of Thrombosis Volume, Anticoagulants, and Recurrence Factors in Portal Vein Thrombosis with Cirrhosis
Source: Life (Basel). 2020 Sep 4;10(9):177. doi: 10.3390/life10090177 (PMC7555202; doi:10.3390/life10090177)
Supplement: Supplementary file 1 [file life-10-00177-s001.pdf]

life

Supplementary

Investigation of Thrombosis Volume, Anticoagulants, and Recurrence Factors in Portal Vein Thrombosis with Cirrhosis

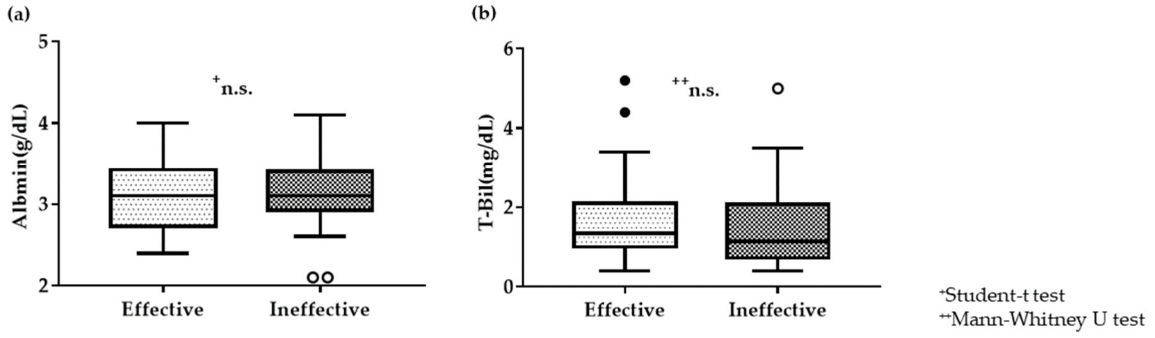

Figure 1. Relationship between correlation factors and PVT reduction.

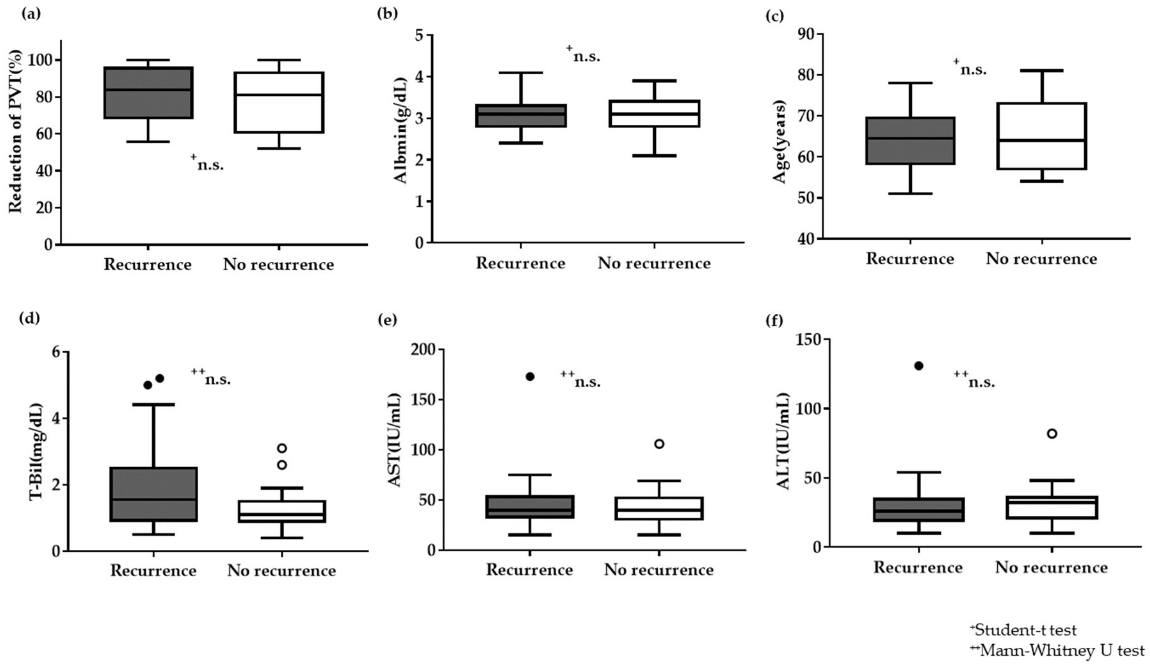

Figure 2. Correlation factors affecting recurrence after anticoagulation therapy.

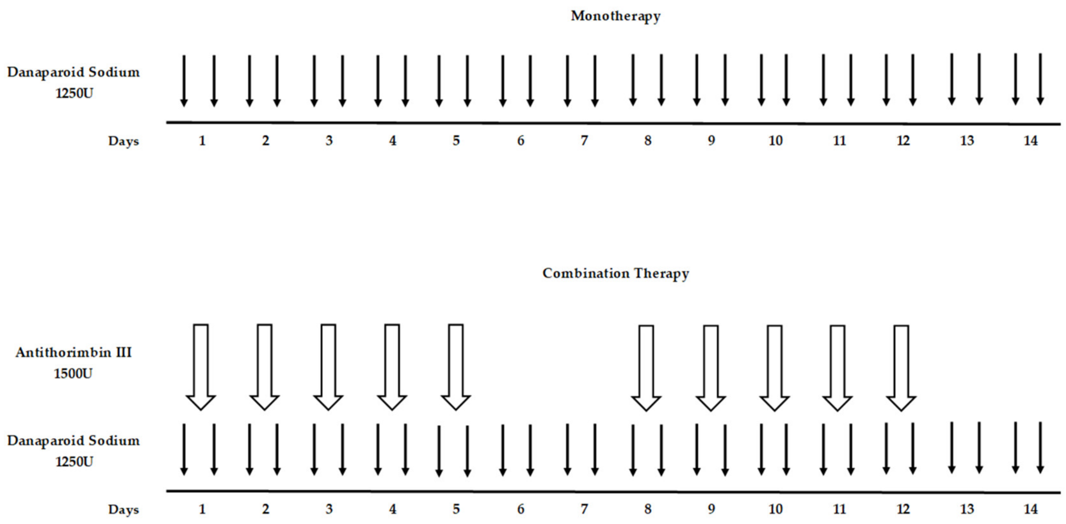

Figure 3. Regimen of danaparoid sodium therapy with and without AT-III anticoagulation therapy.

Table 1. Patients' characteristics in the groups with and without recurrence in effective group.

In effective group	Recurrence (n = 17)	No recurrence (n = 11)	*P*
Child Pugh score	8 (7–12)	7 (6–8)	**n.s.
ALBI score	−1.5 (−1.8 – −1.3)	−1.8 (−2.2 – −1.5)	*n.s
FIB-4 index	6.6 (4.6–10.0)	7.4(2.5–9.5)	*n.s.
Platelet (×10⁴/μL)	8.3 (5.7–12.3)	7.2 (5.5–14.8)	**n.s.
PT activity (%)	67 (57–74)	60 (51–65)	*n.s.
Albumin (mg/dL)	2.9 (2.7–3.2)	3.2 (2.8–3.5)	*n.s.
T-Bil (mg/dL)	1.6 (1.0–2.4)	1.3 (1.0–1.5)	**n.s
AT-III (%)	60 (42.0–71.5)	55 (48.0–69.0)	*n.s.
FDP (μg/mL)	8.5 (6.2–13.7)	8.4 (3.0–17.8)	**n.s.
DD (μg/mL)	4.5 (3.1–8.8)	3.7 (1.0–7.6)	**n.s.
Pretreatment PVT (mL)	3.5 (2.4–6.8)	1.8 (1.2–3.6)	**0.017

Median (IQR); *Student-t test; **Mann Whitney U test.

Table 2. Patients' characteristics in the groups receiving and not receiving maintenance treatment.

	Maintenance (n = 15)	No maintenance (n = 17)	*P*
Child Pugh score	8 (6–10)	7 (6–10)	**n.s.
ALBI score	−1.8 (−2.1 – −1.5)	−1.6 (−2.3 – −1.4)	*n.s.
FIB-4 index	6.6 (3.9–11.0)	6.7 (5.2–9.3)	*n.s.
Platelet (×10⁴/μL)	8.2 (5.5–14.5)	7.3 (4.8–11.5)	**n.s.
AT-III (%)	64 (54–69)	66 (52–73)	*n.s.
Pretreatment PVT (mL)	5.4 (1.9–6.3)	2.6 (1.9–4.5)	**n.s.

Median (IQR); *Student-t test; **Mann Whitney U test

Table 3. Patients' characteristics in the groups receiving monotherapy and combination therapy.

	**Monotherapy (n = 24)**	**Combination therapy (n = 19)**	***P***
Child Pugh score	7 (5, 9)	8 (7, 11)	*0.019
ALBI score	−1.9 (−2.1 – −1.6)	−1.5 (−1.8 – −1.4)	**0.011
FIB-4 index	8.7 (4.0–10.8)	6.6 (5.2–9.0)	*n.s.
Platelet (×10^4/μL)	3.2 (2.9–3.7)	3.0 (2.6–3.2)	**n.s.
PT activity (%)	68 (57–75)	59 (56–67)	*n.s.
Albumin (mg/dL)	3.2 (2.9–3.7)	3.0 (2.6–3.2)	**n.s.
T-Bil (mg/dL)	1.0 (0.4–1.6)	1.6 (1.1–2.6)	**0.019
AT-III (%)	63 (54.3–71.5)	50 (41.0–68.0)	*n.s.
FDP (μg/mL)	8.5 (4.6–21.5)	9.1 (4.4–19.6)	**n.s.
DD (μg/mL)	4.3 (2.0–10.1)	4.3 (2.2–10.9)	**n.s.
Pretreatment PVT (mL)	2.9 (1.7–4.6)	5.4 (2.0–7.6)	**n.s.
Reduction of PVT (%)	81.3 (68.4–93.7)	82.7 (61.2–89.0)	**n.s.
Recurrence (n)	10	14	+n.s.

Median (IQR); *Student-t test; **Mann Whitney U test; +χ2 test

Monotherapy: danaparoid sodium only

Combination therapy: danaparoid sodium with AT-III
